# A comparison of strategies to recruit older patients and carers to end-of-life research in primary care

**DOI:** 10.1186/1472-6963-12-342

**Published:** 2012-09-27

**Authors:** Barbara Hanratty, Elizabeth Lowson, Louise Holmes, Julia Addington-Hall, Antony Arthur, Gunn Grande, Sheila Payne, Jane Seymour

**Affiliations:** 1 Hull York Medical School, Department of Health Sciences, University of York, York, UK; 2 Faculty of Health Sciences, University of Southampton, Southampton, UK; 3 Department of Public Health and Policy, University of Liverpool, Liverpool, UK; 4 School of Nursing, Midwifery and Physiotherapy, University of Nottingham, Nottingham, UK; 5 School of Nursing, Midwifery and Social Work, University of Manchester, Manchester, UK; 6 Division of Health Research, Lancaster University, Lancaster, UK

**Keywords:** Patient selection, Primary health care, Caregivers, Palliative care, Aged, Recruitment to research, End-of-life care research, Research in primary care

## Abstract

**Background:**

Older adults receive most of their end-of-life care in the community, but there are few published data to guide researchers on recruitment to studies in primary care. The aim of this study was to compare recruitment of patients and bereaved carers from general practices in areas with different research network support, and identify challenges in obtaining samples representative of those in need of end-of-life care.

**Methods:**

Comparative analysis of recruitment from general practices to two face-to-face interview studies concerned with 1) carers’ perceptions of transitions between settings for decedents aged over 75 years and 2) the experiences of older patients living with cancer at the end-of-life.

**Results:**

33 (15% of invitees) patients and 118 (25%) carers were interviewed. Carers from disadvantaged areas were under-represented. Recruitment was higher when researchers, rather than research network staff, were in direct contact with general practices. Most practices recruited no more than one carer, despite a seven fold difference in the number of registered patients. The proportion identified as eligible for patient interviews varied by a factor of 38 between practices. Forty-four Primary Care Trusts granted approval to interview carers; two refused. One gave no reason; a second did not believe that general practitioners would be able to identify carers.

**Conclusion:**

Obtaining a representative sample of patients or carers in end-of-life research is a resource intensive challenge. Review of the regulatory and organisational barriers to end-of-life researchers in primary care is required. Research support networks provide invaluable assistance, but researchers should ensure that they are alert to the ways in which they may influence study recruitment.

## Background

End-of-life research presents a range of specific challenges. Deteriorating health for patients and strain on carers limit the desire and the ability to participate in research at the end-of-life. The resulting high rates of attrition and low levels of participation in studies have been widely discussed in the literature [1,2]. Whilst researchers are sensitive and accepting of these limitations on study accrual, the barriers presented by ethical and organisational reviews are more controversial.

The requirement for research to be approved by a Research Ethics Committee and the organisation where the research will be carried out (National Health Service (NHS) research governance in the United Kingdom (UK)) provides important safeguards for patients. These safeguards are in place to protect potentially vulnerable people from being exploited or distressed by research that is intrusive or inappropriate. In common with many other countries, the UK does not allow direct approaches by researchers to patients [3]. Data protection legislation prevents UK NHS organisations from sharing patients’ contact details with researchers without the patients’ permission. Death registration data may be used to identify informants for studies into end-of-life care but all contact must be made through the Office for National Statistics (ONS). A national survey recommended by the End-of-life Strategy in England and Wales was run by ONS, for the first time in 2011/12, for example. But for most projects, initial contact between end-of-life researcher and potential participant must be indirect, via clinicians responsible for a patient’s care or advertisements placed strategically in locations considered visible to the target population [4-6].

As with all safeguards, it is important that protection should be commensurate with the level of risk [7]. In the past, researchers have felt that research ethics committees were too restrictive in denying access to patients [8]. In end-of-life care research, the approach preferred by governance and ethical reviews is generally one where the invited participant must opt-in and contact the researcher to signal their interest in taking part. Opt-in approaches compared with opt-out can easily lead to a reduction in the size and representativeness of a sample [9,10]. Referrals by health professionals into a study may be selective, and judgement on who is a suitable candidate can vary from one doctor or nurse to another [5,6,9,11]. Greater participation in research amongst higher socioeconomic groups is also well recognised [12].

If end-of-life care research is to be methodologically sound and useful to clinicians [13-15], it needs to be conducted in relevant, rather than atypical, settings. Extending studies beyond specialist palliative care is crucial for groups such as older adults who are more likely to be cared for by their general practitioner (GP) [9,13]. The majority of people who receive specialist palliative care have a cancer diagnosis, though they comprise only one in four of all deaths [16]. Almost every person in the UK is registered with a GP, thus there exists the potential for primary care to offer an ideal sampling frame for end-of-life care studies. Ensuring that a study population is representative of different subgroups of the population allows research to contribute to the development of equitable services. Subgroups of race, ethnicity, culture, gender, age, and disease states within the population are known to experience end-of-life care differently, and these differences remain poorly understood [17].

Participant accrual is one of the most important issues in end-of-life care research [18]. There is much discussion of the difficulties in the literature, but few data to guide researchers who are planning end-of-life research in primary care. The practicalities of study planning have tended to be informed by personal experience and advice from colleagues. Estimating how many people a research project may need to approach to attain a certain sample, for example, can be very difficult. Ewing and colleagues sent 1871 letters to yield 36 participants in their primary care study of patients in their last year of life [11]. They felt that gatekeeping by health professionals and ethics committees contributed to their smaller than anticipated sample.

There have been a number of changes since much of the published work on the research process was completed. The processes of applying for NHS Research governance in the UK have recently been centralised. UK data protection law has been strengthened and applications for research ethics committee approval streamlined. In the UK, there has been substantial investment in research infrastructure, with the establishment of research networks in every English region, with staff funded to assist recruitment to approved studies [19].

The aim of this paper is to report on the processes of recruitment to end-of-life studies with carers and patients in three different health regions of England, and identify any common challenges. We compare recruitment in areas where researchers are in direct contact with GPs with those in which research network staff intervene between researchers and GPs.

## Methods and results

Data in this paper are drawn from two separate studies.

### Study one

This study aimed to understand bereaved carers’ perspectives on the end-of-life care experiences of their friend or relative. The focus of enquiry was on movements or transitions between places of care in the last year of life. One hundred and eighteen bereaved carers were recruited and interviewed. They had looked after adults aged over 75 years who died with heart failure, stroke, chronic obstructive pulmonary disease, lung, colorectal or breast cancers. Interviews took place between four and nine months after the death of the care recipient.

### Study two

Study two aimed to explore the experiences of and preferences for care at the end-of-life, amongst older adults in their last year of life. Participants were identified from lists of patients registered with general practitioners. They were eligible if they were in the study age range (75 years and above), had a cancer diagnosis recorded in their medical notes, and if the responsible health professional judged that the patient was in the last year of life. (To refer a patient to the study, the health professional was required to answer ‘no’ to the surprise question: ‘Would you be surprised if your patient was to die within the next twelve months’?) Interviews were conducted with 33 older adults aged over 69 years and living with advanced cancer. A majority (20) lived alone, the rest had co-resident spouse or children.

#### Recruitment of bereaved carers

NHS research governance approval was obtained from 44 NHS Primary Care Trusts (PCTs). Two refused: one felt that the recruitment method would not work, the other gave no reason. A resubmission with more detailed argument to the first PCT was also unsuccessful.

In 107 out of 118 cases, carers were identified by the general practitioners who had looked after the older adult decedent. Eleven carers were recruited through local publicity, or word of mouth amongst health professionals in the study areas.

In all three regions of England, help with identifying general practitioners who were interested in participating, was given by NHS funded research support network. In the two regions in the south of England, the research network staff publicised the study to general practices that had previously identified themselves to the network as being interested and able to participate in research studies. The network staff then put the research team into direct contact with interested GPs. In the third region, network staff publicised the study, liaised with practices, and then visited to search the patient database. They also assisted with sending invitation letters to potential participants.

**Figure 1:**
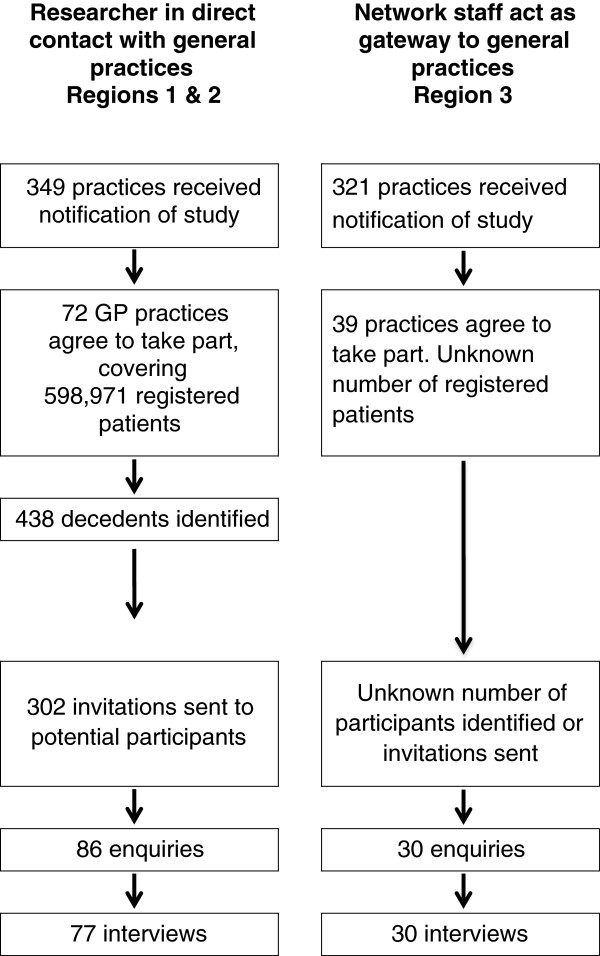
**Recruitment of bereaved carersby general practices withresearch network support.**

Figure 1 compares the recruitment process in the two regions where the researcher was in contact with the GP practices, with the region where network staff played a greater role in the study recruitment. Participants could be excluded if the GP felt that the likelihood of causing distress was high, as well for reasons of ill health or cognitive impairment.

In regions 1 and 2 where detailed data were available on recruitment by GP practice, the number of people interviewed per practice was generally one or none. Three practices identified five or six interviewees. The number of potential participants identified was not related to the number of patients on the GP list. The proportion of people invited who went on to be interviewed, ranged from zero to 100%, with a median of 12.5%, (25^th^ percentile 0%, 50^th^ percentile 12.5%, 75th percentile 33.3%). Table 1 shows the number of carers drawn from areas of different socioeconomic disadvantage, according to the Index of Multiple deprivation. In regions 1 and 2 (southern England), the sample is disproportionately drawn from less disadvantaged areas. In Region 3 (northern England) the sample is more evenly distributed across the quintiles.

**Table 1:** **Carer Interviewees’ Area ofResidence: Number of carersrecruited from areas categorizedby the Index ofMultiple Deprivation**

	**Number (%) of respondents Region 3 (Northern England)**	**Number (%) of respondents Regions 1 & 2 (Southern England)**	**Totals**
**Quintile 1(Most deprived)**	4 (12.5%)	1 (1.2%)	5 (4.4%)
**Quintile 2**	9 (28.1%)	3 (3.7%)	12 (10.6%)
**Quintile 3**	8 (25.0%)	17 (19.8%)	25 (21.2%)
**Quintile 4**	6 (18.8%)	30 (34.9%)	36 (30.5%)
**Quintile 5(Least deprived)**	5 (15.6%)	35 (40.7%)	40 (33.9%)
Totals*	**32 (100%)**	**86 (100%)**	**118(100%)**

Footnote: Note 107 participants were recruited via general practice, the rest by word of mouth, study publicity or other health professionals.

#### Recruitment of patients

Twenty one patients were recruited via their GP practices, into an interview study of adults with advanced cancer aged over 75 years and thought to be in the last year of life. (A further twelve patients were recruited from hospice day care, to ensure the study was completed on schedule). In general practice, the number of patients identified as potential interviewees was unrelated to the size of the practice, or the level of socioeconomic disadvantage with the Primary Care Trust. Two PCTs were amongst the most disadvantaged in England, whilst the other two contained a greater range of socioeconomic experiences (Table 2).

**Table 2:** **Recruitment of Older Patientswith Cancer by GeneralPractices in different PrimaryCare Trusts**

**Area**	**Number of GP practices**	**Number of patients invited**	**Interviews conducted**	**Proportion of interviews frominvitations**
**PCT 1**	3	16	3	18.8%
**PCT 2**	4	14	3	21.4%
**PCT 3**	4	47	8	17.0%
**PCT 4**	4	62	9	14.5%
**Totals**	**15**	**139**	**21**	**15.1%**

The PCTs are listed in order of increasing socioeconomic disadvantage of the population served.

The number of patients identified and invited to participate by each GP practice are shown in Table 2. (Two practices were unable or preferred not to provide this information for the research team). The number of invitees per 100,000 registered patients ranged from 13 to 500, and was unrelated to the method used by the practice to identify potential participants. Approximately half of the reasons stated for excluding patients were clearly related to ill health (Table 3).

**Table 3:** **Recruitment and Reasons forExclusion of Patient Intervieweesby General Practices**

GP list size*	**Identification method**	**Number of patients identified**	**Number of patients invited**	**Invitations per 100,000 patientsregistered**	**Reasons given for exclusion**
3,000	Register of palliative care patients	6	2	66	Cognitive impairment (2), Nursing home residents (2)
3,000	GP knowledge	17	15	500	Cognitive impairment (2)
5,500	Register of palliative care patients	4	4	70	
6,000	Register of palliative care patients	9	9	150	
6,000	Read code search of database	3	3	50	
7,000	Register of palliative care patients	21	11	157	Cognitive impairment (3), Nursing home (1), staying with family (1), emotionally vulnerable (1), near to death (1), does not wish to be invited (1), may live longer than 1 year (2)
7,500	Read code search of database	5	5	66	
8,000	Read code search of database	4	4	50	
9,000	Practice ‘care board’	2	2	22	
11,000	Register of palliative care patients & GP knowledge	16	9	82	No reasons stated
15,000	GP knowledge	2	2	13	
16,000	Register of palliative care patients	9	7	44	‘Too unwell’ (2)
36,000	Read code search of database	49	49	136	

*Data on two practices are not available. List sizes have been rounded to the nearest 500 ensure anonymity.

Two practices listed in Table 3 did provide detailed information about exclusions.

## Discussion

End-of-life care research is sensitive by the nature of the subject matter it seeks to investigate. Researchers working in this area must also consider the role of research networks, research governance authorities and gatekeeping clinicians, when planning studies.

The data in this study provide an insight into the efficiency of recruitment of bereaved carers or older adult patients into interview studies via primary care. The number of interviewees identified per general practice varied widely, and was unrelated to the size of the population from which the interviewees were drawn, or the socioeconomic characteristics of the area in which the practice was located. In the areas where research network staff negotiated between researcher and GP practice, fewer interviews were completed and detailed information on the research process was not readily available. Refusal to grant research governance permission was unpredictable, and in this case, removed an area of high socioeconomic deprivation from the study.

The variation in the number of potential interviewees identified by different practices was so wide, it suggests that there was significant selection going on at that level. This is not surprising in the case of carers, as we did not expect that GPs would be aware of the identity of the carer for all their deceased patients, or that they would necessarily feel comfortable contacting all of the known carers. The practices varied in size from fewer than two thousand to more than fifteen thousand registered patients, yet the majority managed to find at most, one carer to be interviewed. Similarly, the proportion of patients invited to be interviewed varied by a factor of 38 between practices. The carers were drawn disproportionately from less disadvantaged areas. This may reflect the socioeconomic circumstances of the regions involved, but it is possible that GPs selected people who they felt would be better interviewees, because of their education, or availability, or people with whom they had built closer relations during the decedent’s last illness. It is also plausible that people in less deprived areas felt more comfortable about responding to the invitation to be interviewed. We have no data to suggest that the social patterning in the group of carers is related to the way in which research network staff were involved in recruitment.

### Comparison with other work

Data on recruitment are seldom presented in detail, despite this being a fundamental influence on the potential value of research outputs. The response rate of around 25% for carers was lower than some postal surveys of carers with broader inclusion criteria, and other disease-specific studies [20-23]. Ewing and colleagues have previously reported on the barriers presented by professional gatekeeping and NHS governance approvals [11]. When they used a similar opt-in approach to recruiting patients, approximately 1% of invited patients were interviewed. Our higher participation rate (15%) seems most likely to be due to patient related factors, rather than the provision of financial rewards to practices, in the form of modest research support costs. Staff understanding of the study inclusion criteria was not perceived to be a barrier to recruitment [24], though we collected no data to test this assumption.

Low participation rates may be accommodated more readily in qualitative enquiry, when the aim is to extend our understanding of patient and caregiver experiences. However, it would be a serious limitation to any study where obtaining a representative sample was crucial. Qualitative studies with older people near the end-of-life often do not reveal how many people were approached to find their interviewees, and the denominator population is also unknown for studies that recruit via local or national advertising.

### Strengths and limitations

Three different researchers were involved in the work reported here, and we cannot be certain that variation in their tenacity or sensitivity to the clinical context did not contribute to differential recruitment rates. The inclusion and exclusion criteria were specific to our studies, and may have made our recruitment of participants particularly challenging. Both the aim of interviewing carers between four and nine months after the death and the requirement for the decedent to have moved between places of care, limited our population of potential interviewees. Nevertheless, our choice of patients and decedents aged over 75 years captures the majority of deaths in England and Wales, and the conditions specified account for the common causes of predictable death. It is a strength of this analysis that we have information on a large number of participants from qualitative interview studies. And by working across regions, we were able to evaluate the different modes of working with research networks.

### Implications

The nature and extent of gatekeeping by health professionals is an important issue for researchers, as data protection or privacy legislation prevent direct approaches to patients. Gaining the trust of referring clinicians is thought to be important for success [25], and that may be missing when a research network employee acts as an intermediary between researcher and health professional.

The regulatory framework may leave some GPs with difficult judgements to make. Any desire they have to shield vulnerable older adults from potentially unwanted approaches by researchers, must be reconciled with avoiding paternalism, and a policy rhetoric that promotes empowering patients to make their own choices. In the UK, the combination of organisational reform, increasing workloads and greater involvement of private providers in NHS care present a particularly challenging context for researchers in primary care.

The UK Royal College of General Practitioners is currently encouraging its members to identify the 1% of patients on their lists who are likely to die within the following twelve months, in order to improve their care [26]. Even taking into account the fact that our search was restricted to aged patients with cancer, the number identified was considerably lower than could be expected, if every hundredth registered patient is in the last year of life. Identification of people with a short prognosis is still an important barrier to overcome. Financial compensation for general practices who took part in the research was modest, and did not result in a high number of patients being identified. It is possible that payment per participant would be a more effective incentive, though this is not likely to be judged acceptable for end-of-life studies.

People from lower socioeconomic groups or areas are often classified as ‘hard to reach.’ This study provides some data to support that assertion, as the recruited sample of carers was made up of disproportionately fewer people from poorer areas. Allowing sufficient time for purposive recruitment by socioeconomic status may help to overcome this problem, along with raising awareness amongst recruiting health professionals. Without particular attention to social patterning in study recruitment, research has the potential to inadvertently perpetuate inequalities in access to care. NHS authorities play a crucial role in study regulation, and the process by which they allow access to different populations should be transparent and follow nationally agreed criteria.

## Conclusions

In summary, end-of-life research in primary care must often recruit to studies from a denominator population of unknown size, coping with inconsistent application of research governance regulations and gatekeeping by clinicians. In the short term, planning and funding of studies should take this context into account. To resolve these issues and enable investigators to develop robust research, review of the regulatory and organisational barriers to end-of-life researchers in primary care is required.

Ethical approval was granted by Liverpool Central Research Ethics Committee (09/H1005/42) and North West 2 REC (10/H1005/16).

## Competing interests

The authors declare that they have no competing interests.

## Authors’ contributions

BH, EL and LH collated and analysed the data and BH drafted the manuscript. BH, TA, JAH, GG, SP and JS participated in the design of the study. All participated in the interpretation of the analysis, helped to draft the manuscript and read and approved the final manuscript.

## Pre-publication history

The pre-publication history for this paper can be accessed here:

http://www.biomedcentral.com/1472-6963/12/342/prepub
